# Correlation between brain functional connectivity and neurocognitive function in patients with left frontal glioma

**DOI:** 10.1038/s41598-022-22493-6

**Published:** 2022-11-08

**Authors:** Masaya Ueda, Kiyohide Usami, Yukihiro Yamao, Rie Yamawaki, Chinatsu Umaba, Nan Liang, Manabu Nankaku, Yohei Mineharu, Masayuki Honda, Takefumi Hitomi, Ryosuke Ikeguchi, Akio Ikeda, Susumu Miyamoto, Shuichi Matsuda, Yoshiki Arakawa

**Affiliations:** 1 Rehabilitation Unit, Kyoto University Hospital, Kyoto, Japan; 2 Department of Epilepsy, Movement Disorders and Physiology, Kyoto University Graduate School of Medicine, Kyoto, Japan; 3 Department of Neurosurgery, Kyoto University Graduate School of Medicine, Kyoto, Japan; 4 Department of Human Health Sciences, Kyoto University Graduate School of Medicine, Kyoto, Japan; 5 Department of Artificial Intelligence in Healthcare and Medicine, Kyoto University Graduate School of Medicine, Kyoto, Japan; 6 Department of Clinical Laboratory Medicine, Kyoto University Graduate School of Medicine, Kyoto, Japan; 7 Department of Orthopedic Surgery, Kyoto University Graduate School of Medicine, Kyoto, Japan

**Keywords:** Cognitive neuroscience, Computational neuroscience, Electroencephalography - EEG

## Abstract

The association between neurocognitive function (NCF) impairment and brain cortical functional connectivity in glioma patients remains unclear. The correlations between brain oscillatory activity or functional connectivity and NCF measured by the Wechsler Adult Intelligence Scale full-scale intelligence quotient scores (WAIS FSIQ), the Wechsler Memory Scale-revised general memory scores (WMS-R GM), and the Western aphasia battery aphasia quotient scores (WAB AQ) were evaluated in 18 patients with left frontal glioma using resting-state electroencephalography (EEG). Current source density (CSD) and lagged phase synchronization (LPS) were analyzed using exact low-resolution electromagnetic tomography (eLORETA). Although 2 and 2 patients scored in the borderline range of WAIS FSIQ and WMS-R GM, respectively, the mean WAIS FSIQ, WMS-R GM, and WAB AQ values of all patients were within normal limits, and none had aphasia. In the correlation analysis, lower WMS-R GM was associated with a higher LPS value between the right anterior prefrontal cortex and the left superior parietal lobule in the beta1 band (13–20 Hz, R = − 0.802, *P* = 0.012). These findings suggest that LPS evaluated by scalp EEG is associated with memory function in patients with left frontal glioma and mild NCF disorders.

## Introduction

Gliomas are invasive brain tumors that can cause progressive neurocognitive function (NCF) impairment. Comprehensive neurocognitive evaluation in patients with gliomas shows various cognitive deficits, including of attention, memory, language, visuospatial cognition, and executive function^1^. In particular, language-related symptoms such as speech, verbal memory, and verbal working memory disorders in patients with left frontal glioma may emerge through the effects of the glioma on the functional networks of the brain^2–5^. Because these kinds of NCF deficits can significantly impair quality of life (QOL) and social life in patients with gliomas^6,7^, understanding the mechanisms underlying NCF disorders is clinically relevant.

Several studies using functional magnetic resonance imaging (fMRI) and magnetoencephalography (MEG) have recently shown glioma-induced changes in the resting-state functional connectivity of brain networks. These brain networks’ communication changes may be an important tool for predicting the effects and outcomes of perturbations, including lesions and focal stimulation^8^. MEG has high temporal resolution, whereas fMRI has high spatial resolution. Both techniques have shown that gliomas, particularly in the left hemisphere, significantly affect resting brain functional connectivity^9,10^. Furthermore, these changes in brain functional connectivity are correlated with changes in NCF of patients with glioma in the left frontal glioma^11,12^. Previous reports showed that left frontal gliomas cause severe language impairment, although the pattern of language disorders in left frontal gliomas is not as well-understood as in left temporal gliomas, and it is difficult to predict language impairment by assessing structural networks^13,14^. Tumor localization is an important factor that relates to the extent of network disruption, and more homogeneous subgroups of glioma patients are necessary to further explore the clinical potential of non-invasively measured brain activity^13,14^. Furthermore, both modalities are costly and are only used in a small percentage of patients. On the other hand, electroencephalography (EEG) is relatively inexpensive and useful for clinical follow-up.

In the present study, the brain oscillatory activity and functional connectivity of the resting brain were assessed using exact low-resolution electromagnetic tomography (eLORETA)^15^. eLORETA calculates current source density (CSD) as neuronal electrical activity without assuming a specific number of active sources. A method of nonlinear functional connectivity called “lagged phase synchronization” (LPS)^15^, implemented in this eLORETA statistical package, is resistant to non-physiological artifacts, particularly low spatial resolution and the problem of volume conduction^16^. Furthermore, LPS can be used for filtered data, providing frequency decomposition of functional brain connectivity^17^. A study using these methods reported that the mini-mental state examination score correlated with theta band resting-state functional connectivity in patients with Alzheimer’s disease^18^. The physiological significance of frequency activity remains controversial, but it has been suggested that it is related to brain function to some extent^19,20^. For example, theta activity is known to be associated with memory, and alpha activity is associated with inhibition and top-down attention^19^. Previous studies based on electrophysiological measurements of neural activity suggested that different frequency bands are responsible for distinct computational roles^21^, since oscillations are thought to create synchronization across specialized brain regions to corroborate cognitive processing^22^.

The present study investigated the associations between brain oscillatory activity or functional connectivity and NCF in patients with glioma using eLORETA. Only patients with left frontal lobe involvement who were expected to be sensitive to changes in functional connectivity associated with NCF were recruited. It was expected that the brain functional connectivity not only between intra-networks, but also inter-network connectivity, may correlate with NCF. Therefore, we aimed to investigate in an exploratory fashion the correlations between whole-brain cortical oscillatory activity or functional connectivity and NCF in patients with left frontal glioma using resting-state EEG in the present study.

## Results

### Neurocognitive function

The patients’ characteristics are summarized in Table 1. In the Wechsler system, scores of 80 or above are considered normal, meaning low average or better, and the borderline range is 70–79^23,24^. Aphasia is defined based on the WAB AQ cutoff point (93.8)^25^. Results of NCF evaluation are shown in Table 2. Mean (± standard deviation) scores were within normal limits, as follows: FSIQ, 101.24 ± 14.42; GM, 103.83 ± 16.13; and WAB AQ, 98.8 ± 1.32. Two patients scored in the borderline range on the WAIS FSIQ (No. 14, 72; No. 18, 77). Two patients scored in the borderline range on the WMS-R GM (No. 4, 73; No. 14, 78). However, no patients had definitive aphasia based on the WAB AQ score cutoff point.

**Table 1:** Characteristics and Karnofsky performance status scores of patients with left frontal gliomas.

Case	Age (year)	Sex	Pathological diagnosis	WHO grade	Lesion volume (cm^3^)	IDH1	1p/19q codeletion	p53	GFAP	KPS
1	40	F	Oligodendroglioma	2	57.112	M	P	N	–	100
2	44	F	Diffuse astrocytoma	2	21.829	M	N	P	P	100
3	25	M	Oligodendroglioma	2	26.370	M	P	N	P	90
4	29	M	Oligodendroglioma	2	17.963	W	P	N	–	90
5	39	M	Diffuse astrocytoma	2	60.094	M	N	P	–	100
6	37	M	Oligodendroglioma	2	41.825	M	–	N	P	80
7	38	M	Diffuse astrocytoma	2	15.895	M	N	P	P	90
8	24	M	Oligodendroglioma	2	44.199	W	N	N	–	100
9	29	M	Diffuse astrocytoma	2	61.650	M	N	P	P	100
10	35	M	Oligodendroglioma	2	13.506	M	P	N	–	100
11	38	M	Oligodendroglioma	2	44.348	W	P	N	P	100
12	55	F	Diffuse astrocytoma	2	95.787	W	N	N	P	90
13	55	F	Diffuse astrocytoma	2	4.930	M	N	N	P	100
14	37	F	Anaplastic Oligodendroglioma	3	33.742	M	N	N	–	100
15	54	M	Anaplastic astrocytoma	3	160.666	M	N	P	–	90
16	53	F	Anaplastic Oligodendroglioma	3	75.354	M	P	N	P	100
17	72	F	Glioblastoma	4	11.531	W	N	P	P	90
18	63	F	Glioblastoma	4	47.540	W	–	p	–	100

Sex: *F* female, *M* male, *WHO* World Health Organization, *IDH* isocitrate dehydrogenase, *M* mutant, *W* wildtype; 1p/19q codeletion: *P* positive, *N* negative; p53: *P* positive, *N* negative, *GFAP*, glial fibrillary acidic protein, *P* positive, *KPS* Karnofsky performance status.

**Table 2:** Results of evaluations of neurocognitive functions by patient.

Case	WAIS					WMS-R					WAB
	FSIQ	VCI	POI	WMI	PSI	VeM	ViM	GM	A/C	DR	AQ
1	117	120	121	103	84	110	120	114	126	124	99
2	96	104	89	94	102	115	119	118	102	112	100
3	115	106	122	119	102	109	121	114	134	113	98.1
4	NA	NA	NA	NA	NA	66	104	73	95	69	98.4
5	107	105	108	102	100	102	120	107	103	110	100
6	107	100	125	107	94	87	115	94	132	105	99.6
7	87	NA	NA	NA	NA	74	118	84	91	94	100.0
8	100	92	114	103	89	109	105	99	94	114	99.2
9	96	107	93	81	75	115	104	113	89	99	100.0
10	110	116	103	98	97	116	104	114	92	97	99.6
11	105	111	114	85	84	97	101	98	104	98	97.7
12	72	85	85	67	68	68	108	78	76	85	98.5
13	116	110	124	114	105	104	113	108	114	114	100
14	114	107	128	109	102	110	122	115	120	120	98.9
15	80	77	97	76	82	106	108	107	92	90	96.8
16	109	115	124	100	79	132	119	132	102	127	100
17	113	110	103	117	118	123	105	119	123	118	96.8
18	77	99	87	58	78	82	90	82	94	82	95.7
Av	101	104	109	96	91	101	110	104	105	104	98.9
SD	14.4	11.4	15.0	17.8	13.4	18.8	8.9	16.1	16.5	15,8	1.32

WAIS scores: *FSIQ* full-scale intelligence quotient, *VCI* verbal comprehension index, *POI* perceptual organization index, *WMI* working memory index, *PSI* processing speed index; WMS-R scores: *Verbal memory* verbal memory, *Visual memory* visual memory, *GM* generalized memory, *A/C* attention concentration, *DR* delayed recall; WAB: *AQ* aphasia quotient, *Av* Average of all patients, *SD* standard deviation. In the Wechsler system, scores of 80 or above are considered normal, meaning low average or better, and the borderline range is 70–79^23,24^. Aphasia is defined based on the WAB AQ cutoff point (93.8)^25^.

### Associations between CSD and evaluation of neurocognitive function scores

**Figure 1:**
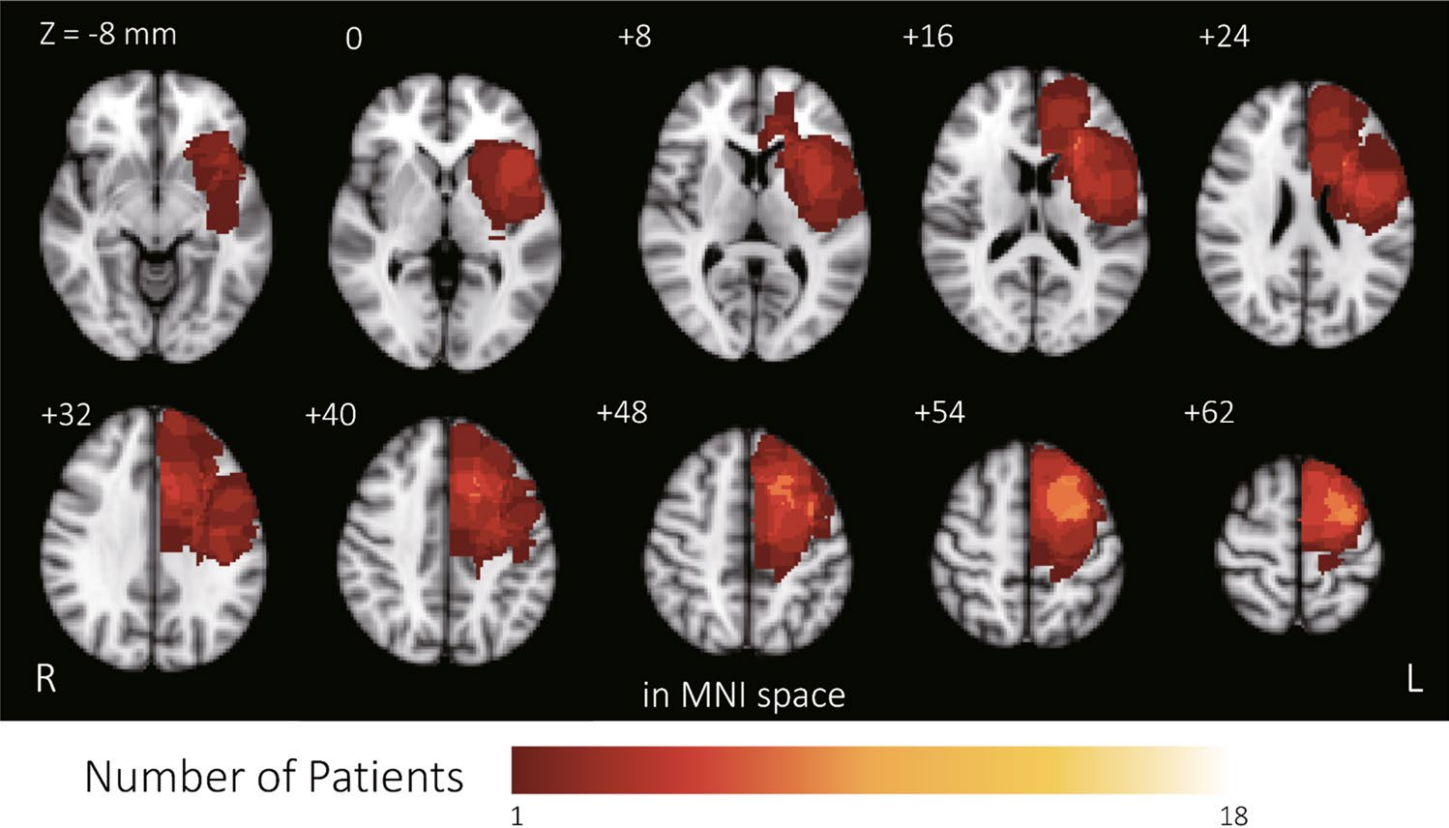
Lesion topography of 18 patients with left frontal gliomas in Montreal Neurological Institute space. Each voxel was identified as part of the tumor region from at least one patient. The color bar represents the number of patients with a lesion on a specific voxel.

**Figure 2:**
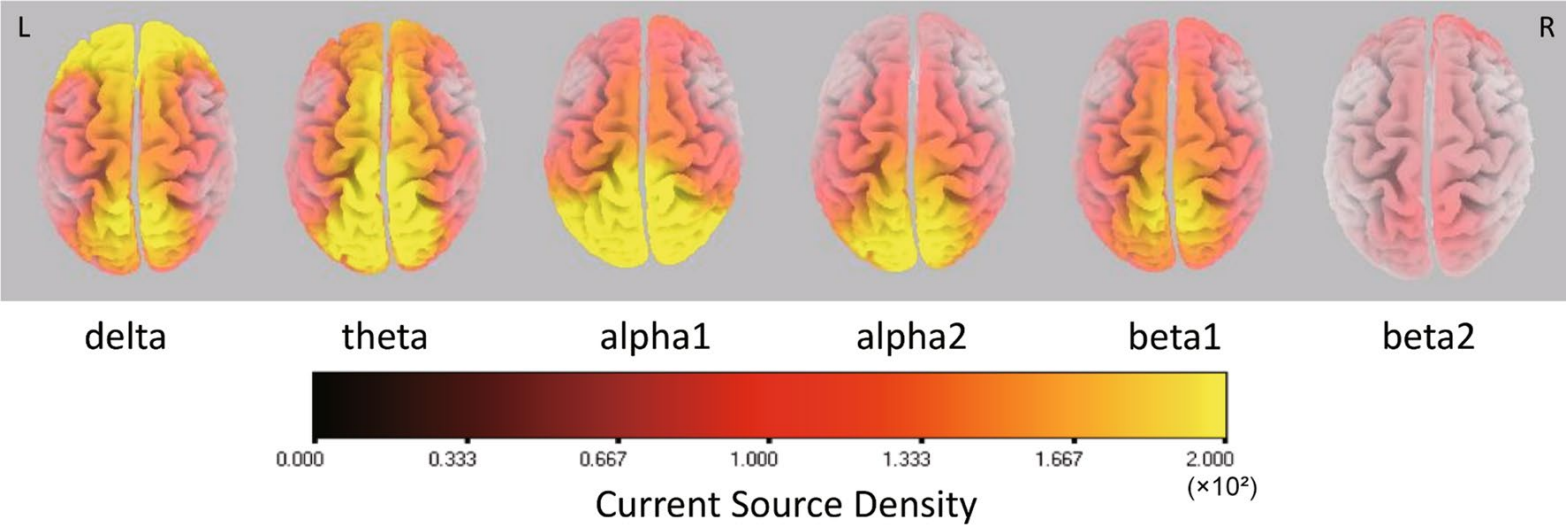
Averaged eLORETA source current density in each frequency band in patients with left frontal gliomas. The color map indicates the current source density (CSD) from black at 0 to yellow at 2.0.

Lesion topography of 18 patients with left frontal gliomas in the Montreal Neurological Institute (MNI) space was created by stacking and binarizing tumor masks of all subjects (Fig. 1). Averaged CSD by eLORETA in each frequency band was calculated in patients with left frontal gliomas (Fig. 2). The highest CSD values were found in the alpha1 band in the left cuneus of the occipital lobe (CSD value, 366), followed by the delta band at the medial frontal gyrus (CSD value, 336). The associations between CSD values and FSIQ, GM, and AQ were investigated in patients with left frontal gliomas. All 6,239 cortical voxels were explored in the CSD analysis. The CSD analysis showed no significant correlations between CSD and all cognitive examination scores. As a supplementary analysis, the correlations between FSIQ, GM, and AQ and log CSD were explored. This analysis also showed no significant correlations between log CSD and evaluations of NCF.

### Association between LPS and neurocognitive function scores

To analyze functional connectivity, a voxel-wise approach was adopted to determine cortical regions of interest (ROIs). With eLORETA, MNI coordinates of the cortical voxels underlying the electrode sites were defined to create the ROIs. Based on previous papers on functional connectivity by means of EEG^18^ and fMRI^26^, 21 ROIs were selected. Three additional ROIs comprising two auditory fields and visual fields were included, since they have recently attracted attention in studies on brain functional networks^27,28^. As a result, a total of 24 cortical ROIs were defined (Table 3).

**Table 3:** The 24 cerebral regions of interest determined by eLORETA.

Anatomical region	Brodmann area	ROI centroid MNI coordinates		
		*x*	*y*	*z*
Left middle temporal area (lMT)	37	− 50	− 70	− 5
Right middle temporal area (rMT)	37	45	70	− 5
Left frontal eye fields (lFEF)	6	− 25	− 10	50
Right frontal eye fields (rFEF)	6	25	− 10	50
Left superior parietal lobule (lSPL)	7	− 25	− 50	55
Right superior parietal lobule (rSPL)	7	25	− 50	55
Left anterior prefrontal cortex (laPFC)	10	− 35	55	5
Right anterior prefrontal cortex (raPFC)	10	35	55	5
Left dorsolateral prefrontal cortex (ldlPFC)	9	− 50	15	40
Right dorsolateral prefrontal cortex (rdlPFC)	9	50	15	40
Anterior cingulate cortex (aCC)	32	5	30	25
Left anterior inferior parietal lobule (laIPL)	40	− 50	− 50	45
Right anterior inferior parietal lobule (raIPL)	40	50	− 50	45
Left anterior insula (laINS)	47	− 30	20	− 5
Right anterior insula (raINS)	47	30	20	− 5
Left hippocampal formation (lHF)	28	− 20	− 20	− 20
Right hippocampal formation (rHF)	28	20	− 20	− 20
Ventromedial prefrontal cortex (vmPFC)	10	− 5	50	− 5
Posterior cingulate cortex (pCC)	23	0	− 55	15
Left posterior inferior parietal lobule (lpIPL)	39	− 50	− 70	30
Right posterior inferior parietal lobule (rpIPL)	39	50	− 70	30
Visual fields (Vis)	18	0	− 90	− 5
Left auditory fields (lAud)	41	− 55	− 25	10
Right auditory fields (rAud)	41	55	− 25	10

*ROI* region of interest.

**Figure 3:**
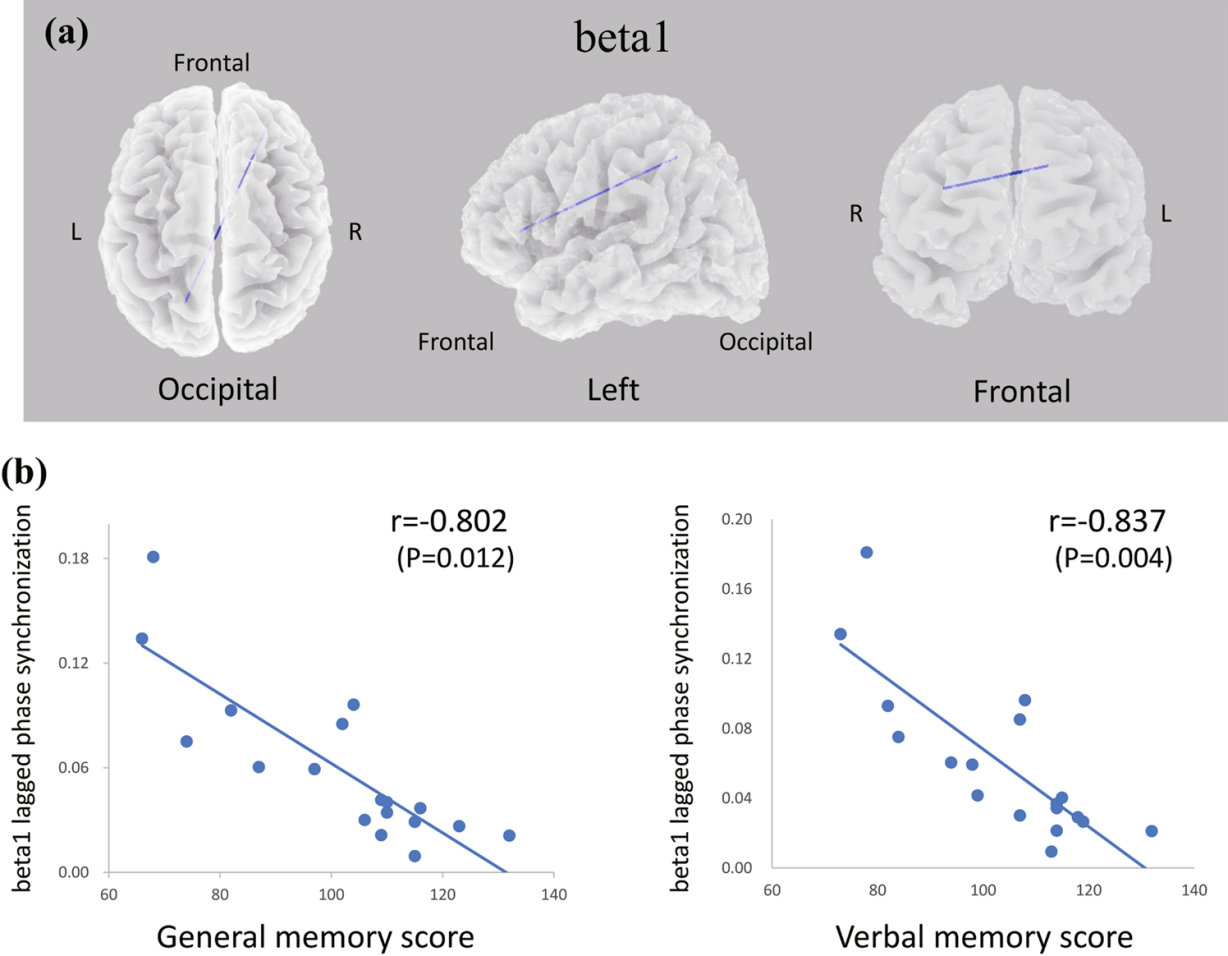
Association between lagged phase synchronization and general memory and verbal memory scores by eLORETA. (**a**) eLORETA wire diagrams of the right anterior prefrontal cortex (raPFC) and left superior parietal lobule (lSPL) show significant negative correlations with general memory (GM) and verbal memory scores in beta1 lagged phase synchronization (LPS). The blue line indicates connectivity between raPFC and lSPL, with a significant negative correlation between GM and verbal memory scores and beta1 LPS. (**b**) Scatter plots of GM and beta1 LPS values, verbal memory scores and beta1 LPS value intensities for 18 subjects. GM and verbal memory scores show strong negative correlations with the LPS value between raPFC and lSPL in the beta1 band (GM score R = − 0.802, *P* = 0.012; Verbal memory score R = − 0.837, *P* = 0.004).

The associations between LPS values of all pairwise possibilities between ROIs and FSIQ, GM, and AQ were analyzed. The analysis showed a significant negative correlation between GM and functional connectivity in the beta1 band (Fig. 3). GM showed a strong negative correlation with the LPS value between the right anterior prefrontal cortex (raPFC) and the left superior parietal lobule (lSPL) in the beta1 band (R = − 0.802, *P* = 0.012); the lower the GM, the higher the LPS values between these regions. In addition, the correlations between the group index scores of WMS-R (verbal memory, 101.39 ± 18.79; visual memory, 110.89 ± 8.94; attention/concentration, 104.61 ± 16.51; delayed memory, 103.94 ± 15.79) scores and LPS values were analyzed. Post hoc analysis also showed that the verbal memory score had a negative correlation with LPS between raPFC and lSPL in the beta1 band (R = − 0.837, *P* = 0.004) (Fig. 3).

## Discussion

In the present study, we investigated the associations between resting-state EEG parameters (i.e., CSD and LPS values) and NCF in patients with gliomas in the left frontal lobe. Two patients showed a low FSIQ, and two patients had memory impairment. Nevertheless, mean FSIQ and GM values were within normal limits (FSIQ ranged from 86.62 to 115.66 and GM 87.7 to 119.96)^29,30^, and no patients were under the cutoff point of AQ (93.8)^25^. GM and verbal memory scores showed strong negative correlations with the LPS value between raPFC and lSPL in the beta1 band (13–20 Hz). Patients with left frontal gliomas have often shown some language function impairment^1^. However, the present results for the WAIS and WMS-R indices showed that nearly all patients performed within normal limits, and none exhibited significant aphasia based on WAB AQ. Resting-state fMRI and MEG studies have shown that compensation, such as increased functional connectivity in the cerebellum and language-related regions of the right hemisphere, occurs in patients with left-brain glioma^31,32^.

Analyses of associations between CSD and NCF scores showed no significant correlations. The characteristics and distribution of electroencephalographic changes produced by a tumor depend primarily on the size of the lesion, its rate of growth, its distance from the cortical surface, and the tumor type^33^. In particular, when brain tumors are located superficially, focal slow waves can be observed on scalp EEG^33^. In the present study, the tumor sizes of left frontal lobe gliomas were heterogeneous, and the distance from the brain surface and the location of the lesion varied among patients. Nevertheless, most of the electroencephalographic data used in the analysis did not include slow activities that indicate localized abnormalities. Thus, CSD of the present subjects included fewer individual differences, which may be a reason why no correlations with NCF were identified. This research indicated that GM and the verbal memory score were inversely correlated with functional connectivity between the raPFC and lSPL. The GM was constructed by the sum of the verbal memory and visual memory scores. The post hoc analysis showed that this functional connectivity was more related to verbal memory than visual memory. Recent studies have shown that changes in functional connectivity are associated with changes in NCF in glioma patients^34,35^. The present results differed in that the correlation was negative. Brain functional connectivity is pathologically enhanced by aging and neurological diseases and is associated with worsening symptoms and NCF decline^36–38^. Lockhart showed that cognitive performance in healthy older adults was inversely correlated with increased functional connectivity in the frontal lobes and argued that this result was a “toxic consequence” rather than a compensatory connection^38^. In fact, several studies have suggested that increased connectivity reduces the efficiency of information transfer and does not ultimately aid in NCF^39,40^. The negative correlations observed in the present study may also reflect such pathological changes.

Another significant aspect of the results is that the functional connectivity associated with verbal memory extended to the contralateral hemisphere. Resting-state fMRI and MEG studies in patients with gliomas have indicated that inter-network connections were stronger than in healthy controls and were often observed in unexpected pairs and regions^12,17^. Previous studies of the function of fronto-parietal connectivity have reported that the connectivity between the medial aPFC and the right central precuneus and intraparietal sulcus/inferior parietal lobule was associated with metacognitive ability for memory retrieval^41^. It also emphasizes the relationship between parietal-frontal connectivity and impaired updating and manipulation of contextual information and delusions, mainly in patients with schizophrenia^42,43^. These findings of connectivity between the right anterior prefrontal cortex and the left superior parietal lobe are associated with memory. The present results suggest that left frontal lobe glioma possibly has some deleterious effect on this memory-related functional connectivity. However, direct comparisons should be made with caution due to the quite different patient groups involved. Several previous studies have reported that functional connectivity in the resting alpha band is linearly associated with improvable misorients in language and NCF in stroke patients and glioma patients^44–46^. The result of the present study was unexpected, since it is generally known that beta activity at rest is associated with sensory-motor function^47^. Although the physiological meaning of frequency bands remains controversial, several studies have suggested that beta band activity is involved in mediating spatially distributed brain networks, such as those between the temporal and parietal lobes^48,49^. In particular, it has been pointed out that beta1 band activity mediates between the wide range of brain regions necessary for multimodal (multisensory integrated) information processing^38^. The present results may reflect these characteristics of the beta1 band.

Tumor location has been reported to be related to the severity and types of NCF, 1 and a frontal glioma in the dominant hemisphere is known to cause severe NFC disorders^2,3^. However, the patients in the present study had less NCF impairment than in previous studies. This is because 13 patients (72.2%) had WHO grade 2 glioma, and 3 patients (16.7%) had WHO grade 3 glioma with isocitrate dehydrogenase 1 (IDH1) mutation, which progress slowly. Thus, the present finding might be related to the network changes associated with NCF impairment in patients with slowly progressing gliomas. Fox mentioned in his review article that gliomas lead to various alterations in functional connectivity across different functional networks, and the patterns have potential as biomarkers of NCF outcomes^50^. The present findings, such as those from scalp EEG data, are clinically useful and suggest that assessment of brain functional connectivity may be important in the search for biomarkers of slowly progressing gliomas.

There are some limitations to the present research. First, although the locations of the lesions were relatively similar, the World Health Organization grade of tumor (13 patients with low-grade tumor, 5 patients with high-grade tumor), tumor size, and degree of progression may have differed. In patients with glioblastoma, slow waves are more likely to be seen in lesions closer to the brain surface^50^. Nevertheless, little abnormal activity was observed in their EEG data, and their KPS scores were over 90. Thus, the effect on the present study would have been minimal. In addition, previous studies have reported that the biomolecular effect of glioma, including IDH1 mutation status, has a strong possibility of progressing to NCF decline in patients with glioma^51–53^. In the present study, 13 patients (72.2%) had WHO grade 2 gliomas, and 3 patients (16.7%) had WHO grade 3 gliomas with IDH1 mutation, which progress slowly. The number of subjects was not large enough to perform an independent analysis for each group. Therefore, the present study may have missed some important results due to differences in IDH status. Second, since the number of electrodes was small (19 channels), discussion of localization requires careful interpretation. In the present study, eLORETA was used to analyze brain oscillatory activity and functional connectivity to resolve problems of spatial resolution. However, using eLORETA for exploring brain function has methodological limitations. For example, despite the proven utility and reproducibility, eLORETA results rely on an inferred model and may not accurately represent the origin of brain activity. In addition, artifacts and non-resting conditions prevented multiple random sampling of the clinical EEG data, so that test–retest reliability could not be established in the present study. Third, in the present study, no control population was included; therefore, the effects of the glioma, relative to a general population, were not assessed. Thus, it was impossible to determine whether the functional connectivity was normal or abnormal in comparison with healthy controls. Fourth, the present study showed less neurocognitive impairment using WAIS and WMS composite indices than previous similar studies. However, these composite indices for solving the problem of multiple comparisons may “washout” impairment at the subtest level. Furthermore, the measures used do not comprehensively sample all cognitive domains. Specifically, executive functioning is not well-represented in the IQ or memory batteries. Further studies are needed to characterize the NCF and functional brain connectivity of patients with gliomas, such as through comparisons of controlled tumor location grades and matched control subjects.

## Conclusions

The evaluation of NCF with WAIS FSIQ, WMS-R GM, and WAB AQ found NCF disorders in a small number of patients with left frontal gliomas enrolled in the present study. In addition, analysis of NCF and brain functional connectivity demonstrated a correlation between general and verbal memory and functional connectivity in the beta1 band of the right anterior prefrontal cortex and the left superior parietal lobule. The associations observed more properly indicate a general relationship between LPS and memory function, but not necessarily impairment itself. These findings suggest that LPS evaluated by scalp EEG is associated with memory function in patients with left frontal gliomas and mild NCF disorders.

## Methods

### Ethics approval, guidelines, and consent to participate

This was a cross-sectional study. The ethics committee of Kyoto University Graduate School and Faculty of Medicine (R1515) approved this study protocol. All procedures were performed in accordance with the Declaration of Helsinki. This study conforms to all Strengthening the Reporting of Observational Studies in Epidemiology guidelines. Written, informed consent was obtained from all patients prior to participation. Patients with a history of brain injury or drug/alcohol abuse were excluded from this study.

### Patients

The participants were 18 patients (mean age, 43 ± 13.15 years) with newly diagnosed glioma in the left frontal lobe who were treated in Kyoto University Hospital and underwent neuropsychological examination and EEG between April 2010 and March 2021. The inclusion criteria were as follows: (1) tumor located in the left frontal lobe; (2) tumor pathology confirmed as glioma by surgery; (3) Karnofsky performance status (KPS) score > 70 (slight disability: unable to conduct all previous activities, but able to look after own affairs without assistance); (4) no or only mild neurological focal deficit, such as speech disturbance or paresis; and (5) at least 12 years of education. Tumor pathology was confirmed at the time of surgical removal of the tumor. In the present study, 6 patients had IDH1 wild type gliomas, and 12 patients had IDH1-mutant gliomas^54^. Tumor masks of all subjects in Montreal Neurological Institute (MNI) space were then stacked and binarized to construct a tumor-overlapping image using the FMRIB Software Library (www.fmrib.ox.ac.uk/fsl)^55^ (Fig. 1), in which each voxel was identified as part of the tumor region from at least one patient. The Edinburgh handedness inventory was used to confirm that all patients were right-handed. All patients were on antiseizure medication for control or prevention of epilepsy.

### Evaluation of neurocognitive function

NCF assessment took place before surgery or before biopsy preceding resection surgery. A total of 18 patients finished evaluation of NCF before starting treatment for glioma. All NFC assessment methods and norms used in the present study were Japanese versions, validated, and age-corrected. NCF was investigated in patients with glioma using the Wechsler Adult Intelligence Scale-revised (WAIS-R, n = 1), WAIS-III (n = 9), or WAIS-IV (n = 7), Wechsler Memory Scale-revised (WMS-R), and original Western Aphasia Battery (WAB). The subtests (excluding supplementary scales) were performed on all patients. WAIS and WMS are comprehensive neuropsychological evaluation instruments with high reliability and validity for detecting minimal changes in NCF^56,57^. One subject (Case 4) could not finish the WAIS due to a lack of time available for testing. The FSIQ of another patient (Case 7) was measured using the WAIS-R, and thus the WAIS group index scores (VCI, POI, WMI, PSI) were not calculated. In the Wechsler system, scores of 80 or above are considered normal, meaning low average or better, and the borderline range is 70–79^23,24^. Aphasia is defined based on the WAB AQ cutoff point (93.8)^25^.

### EEG recordings and data acquisition

EEG recordings took place before surgery or before biopsy preceding resection surgery. All patients underwent clinical EEG recording for 30 min to identify paroxysmal discharges, since patients with glioma often have epilepsy^58^. In the present study 40 s of artifact-free resting-state data from that EEG data from the 18 patients with left frontal gliomas were analyzed. Only data obtained during the resting state with eyes closed and in an arousal state were used. A certified clinical neurophysiologist (K.U.) checked the findings and confirmed that interictal epileptic discharges or continuous focal slow waves were excluded from the analysis windows.

EEGs were recorded with a digital 19-channel scalp EEG device, using the International 10–20 system (i.e., Fp1, Fp2, F7, F3, Fz, F4, F8, T7, C3, Cz, C4, T8, P7, P3, Pz, P4, P8, O1, O2). EEG data were acquired with a linked ear reference, sampled at 500 Hz, and filtered off-line between 0.53 and 60 Hz. Electrode impedance was kept below 5 kΩ. EEG recording included eyes open and closed states with vigilance control. For all patients, 40 s of artifact-free EEG data, fragmented off-line into 2-s segments, were selected. EEG findings were clinically analyzed by certified electroencephalographers. The EEG data used for the following analyses excluded findings suggestive of focal abnormality (focal slow waves) or interictal epileptic activities (spike or sharp waves). Segments including artifacts generated by blink or muscle movements, or signs of drowsiness were rejected, and only reliable, awake EEG data were selected to ensure that brain function during the resting-state could be adequately evaluated. EEG data were analyzed using the LORETA-KEY software package (product by The KEY Institute for Brain-Mind Research).

At least 2 s of data from continuous artifact-free EEG recordings as one epoch is required for eLORETA analyses. To avoid behavioral and EEG drowsiness, a skilled experimenter monitored the patient, and in the event of apparent EEG drowsiness, verbal instructions and warnings were provided. EEGs that suggested drowsiness were rejected in data processing. EEGs contaminated with ocular and muscular artifacts were also rejected. These activities usually exhibited an amplitude of more than 100 μV. However, ocular and muscular artifacts less than 100 μV were also excluded to the extent possible when some activity was considered likely to represent such artifacts based on waveforms and distributions. The epochs that included sporadic slow waves were excluded for exploring the steady resting state.

### EEG source localization

The cortical distribution of the current source density (CSD) was investigated using eLORETA. The head model in eLORETA and the electrode coordinates are based on the MNI average MRI brain map (MNI152)^59^. The solution space was limited to the cortical gray matter, including 6239 voxels with a spatial resolution of 5 mm. Previous studies have validated eLORETA tomography using fMRI^60,61^, structural MRI^62^, positron emission tomography (PET)^63^, and intracranial EEG^26^. In the present study, intracerebral electrical sources that yielded scalp-recorded potentials in each frequency band were estimated using CSD values. Selected artifact-free EEG fragments were analyzed to calculate the eLORETA cortical CSD from 0.53 to 30 Hz. CSD of the eLORETA cortical functioning image was calculated for 6 frequency bands: delta, 2–4 Hz; theta, 4–8 Hz; alpha1, 8–10 Hz; alpha2, 10–13 Hz; beta1, 13–20 Hz; and beta2, 20–30 Hz.

### Functional connectivity analysis

Although detailed information on the eLORETA connectivity algorithm has recently been published elsewhere^15,64^, this method is briefly summarized. LPS was used to analyze functional connectivity between all pairs of ROIs. This is a method for evaluating similarities between signals in the frequency domain, based on normalized Fourier transforms. LPS is thus associated with nonlinear functional connectivity. This lagged connectivity measure is considered to be accurately corrected because it represents the connectivity of two signals after excluding the instantaneous zero-lag component (i.e., several artifact elements). Such correction is necessary because scalp EEG signals or estimated intracranial signals (EEG tomography) often include non-physiological components or physical artifacts, such as volume conduction, that usually affect other connectivity indices. LPS is thus considered to include only physiological connectivity information.

### Statistical analyses

The associations between EEG parameters (i.e., CSD values of all voxels and LPS values of all pairwise possibilities between regions) and NCF scores were assessed by performing regression analysis using the eLORETA software. For the statistical analysis, eLORETA applied a statistical nonparametric mapping method (SnPM). First, the WAIS full-scale intelligence quotient (FSIQ) score, WMS-R generalized memory (GM) scores, and WAB aphasia quotient (AQ) were used for this statistical analysis. In addition, the correlations between the group index scores in WAIS or WMS-R and the EEG parameters were also analyzed when significant correlations were detected between the EEG parameters and these indices of NCF.

The critical probability threshold for *P* values was set at *P* = 0.05 and determined by an SnPM with correction for multiple comparisons across all frequencies. The use of SnPM for LORETA images has been validated in several studies^29,65^. In the resulting statistical three-dimensional images, cortical voxels or functional connectivity between ROIs showing significant differences were identified by a nonparametric randomization/permutation procedure. By estimating the empirical probability distribution for the “maximal-statistic” under the null hypothesis, randomization and permutation tests have proven to be effective in controlling the family-wise error rate (Type I error) in neuroimaging studies^30^. eLORETA used 5000 data randomizations to determine the critical probability threshold values with correction for multiple comparisons across all voxels or functional connectivity and all frequencies, without the need to rely on Gaussianity^66^. Details on the nonparametric randomization procedure are provided in the study by Nichols and Holmes^30^. Furthermore, to correct for multiple comparisons, the eLORETA non-parametric randomization procedure based on the “maximal statistic” was used^30^. The omnibus null hypothesis was rejected if at least one *t* value was above the critical threshold for *P* = 0.05 determined by 5000 data randomizations.

In the further step, the results of correlation analysis for the NCF scores in multiple tests were corrected using the Bonferroni method. Specifically, the Bonferroni method was used to determine the adjusted significance level (*P* < 0.05/3 = 0.017) for the correlation test results for each analysis.

## Supplementary Information

Supplementary Table 1.

## Data Availability

The datasets used and/or analyzed during the present study are available from the corresponding author on reasonable request.

## Abbreviations

AQ: Aphasia quotient
CSD: Current source density
EEG: Electroencephalography
eLORETA: Exact low-resolution electromagnetic tomography
fMRI: Functional magnetic resonance imaging
FSIQ: Full-scale intelligence quotient
GFAP: Glial fibrillary acidic protein
GM: General memory
IDH1: Isocitrate dehydrogenase 1
KPS: Karnofsky performance status
LPS: Lagged phase synchronization
lSPL: Left superior parietal lobule
MEG: Magnetoencephalography
MNI: Montreal Neurological Institute
NCF: Neurocognitive function
PET: Positron emission tomography
QOL: Quality of life
raPFC: Right anterior prefrontal cortex
ROI: Region of interest
WAB: Western aphasia battery
WAIS: Wechsler Adult Intelligence Scale
WHO: World Health Organization
WMS-R: Wechsler memory scale-revised
