# Supplementary material for: Correlation between brain functional connectivity and neurocognitive function in patients with left frontal glioma
Source: Sci Rep. 2022 Nov 8;12:18302. doi: 10.1038/s41598-022-22493-6 (PMC9643499; doi:10.1038/s41598-022-22493-6)
Supplement: Supplementary file 1 — Supplementary Table 1. [file 41598_2022_22493_MOESM1_ESM.docx]

**Correlation between brain functional connectivity and neurocognitive function in patients with left frontal glioma**

Masaya Ueda*^1^, Kiyohide Usami^2^, Yukihiro Yamao^3^, Rie Yamawaki^1^, Chinatsu Umaba^1,4^, Nan Liang^4^, Manabu Nankaku^1^, Yohei Mineharu^3,5^, Masayuki Honda^6^, Takefumi Hitomi^6^, Ryosuke Ikeguchi^1,7^, Akio Ikeda^2^, Susumu Miyamoto^3^, Shuichi Matsuda^1,7^, Yoshiki Arakawa*^3^

1. Rehabilitation Unit, Kyoto University Hospital, Kyoto, Japan
2. Department of Epilepsy, Movement Disorders and Physiology, Kyoto University Graduate School of Medicine, Kyoto, Japan
3. Department of Neurosurgery, Kyoto University Graduate School of Medicine, Kyoto, Japan
4. Department of Human Health Sciences, Kyoto University Graduate School of Medicine, Kyoto, Japan
5. Department of Artificial Intelligence in Healthcare and Medicine, Kyoto University Graduate School of Medicine, Kyoto, Japan
6. Department of Clinical Laboratory Medicine, Kyoto University Graduate School of Medicine
7. Department of Orthopedic Surgery, Kyoto University Graduate School of Medicine, Kyoto, Japan

**Running title:** Brain functional connectivity and neurocognitive function in left frontal glioma

***Corresponding author:**

Masaya Ueda, Rehabilitation Unit, Kyoto University Hospital, 54 Shogoin Kawahara-cho, Sakyo-ku, Kyoto 606-8507, Japan

Tel: +81-75-366-7729

E-mail: ueda0709@kuhp.kyoto-u.ac.jp

Yoshiki Arakawa, Department of Neurosurgery, Kyoto University Graduate School of Medicine, 54 Shogoin Kawahara-cho, Sakyo-ku, Kyoto 606-8507, Japan

Tel: +81-75-751-3459

E-mail: yarakawa@kuhp.kyoto-u.ac.jp

**Supplementary Table.** *P*-value of correlation analysis between WAIS, WMS, and WAB group index scores and connectivity measures by eLORETA

| W | WAIS | | | | | | WMS-R | | | | | |  | | WAB | | | |
| --- | --- | --- | --- | --- | --- | --- | --- | --- | --- | --- | --- | --- | --- | --- | --- | --- | --- | --- |
|  |  | FSIQ | VCI | POI | WMI | PSI | |  | VeM | ViM | GM | A/C | | DR | |  | AQ |  |
| CSD |  | 0.686 | 0.199 | 0.617 | 0.414 | 0.953 | |  | 0.980 | 0.387 | 0.954 | 0.805 | | 0.571 | |  | 0.123 |  |
|  |  |  |  |  |  |  | |  |  |  |  |  | |  | |  |  |  |
| LPS |  | 0.620 | 0.705 | 0.269 | 0.604 | 0.276 | |  | 0.004 | 0.895 | 0.012 | 0.830 | | 0.787 | |  | 0.817 |  |
